# The effect of Ramadan fasting on fetal development

**DOI:** 10.12669/pjms.316.8562

**Published:** 2015

**Authors:** Atilla Karateke, Mustafa Kaplanoglu, Fazil Avci, Raziye Keskin Kurt, Ali Baloglu

**Affiliations:** 1 Dr. Atilla Karateke, Antakya State Hospital of Obstetrics and Child Care, Hatay, Turkey; 2 Dr. Mustafa Kaplanoglu, Department of Obstetrics and Gynecology, Adiyaman University Medical School, Adiyaman, Turkey; 3 Dr. Fazil Avci, Agri Patnos State Hospital of Obstetrics and Child Care, Agri, Turkey; 4 Dr. Raziye Keskin Kurt, Department of Obstetrics and Gynecology, Mustafa Kemal University Medical School, Hatay, Turkey; 5 Dr. Ali Baloglu, Izmir Private Gynecology Clinic, Izmir, Turkey

**Keywords:** Ramadan, Fasting, Fetal development

## Abstract

**Objective:**

To evaluate the effects of Ramadan fasting on fetal development and outcomes of pregnancy.

**Methods:**

We performed this study in Antakya State Hospital of Obstetrics and Child Care, between 28 June 2014 and 27 July 2014 (during the month of Ramadan). A total of two hundred forty healthy pregnant women who were fasting during Ramadan, were included in the groups. The three groups were divided according to the trimesters. The each group was consisted of 40 healthy pregnant women with fasting and 40 healthy pregnant women without fasting. For evaluating the effects of Ramadan on fetus, ultrasonography was performed on all pregnant women in the beginning and the end of Ramadan. We used the essential parameters for the following measurements: increase of fetal biparietal diameter (BPD), increase of fetal femur length (FL), increase of estimated fetal body weight (EFBW), fetal biophysical profile (BPP), amniotic fluid index (AFI), and umbilical artery systole/diastole (S/D) ratio.

**Results:**

No significant difference was found between the two groups for the fetal age, maternal weight gain (kilogram), estimated fetal weight gain (EFWG), fetal BPP, AFI, and umbilical artery S/D ratio. On the other hand, a statistically significant increase was observed in maternal weight in the second and third trimesters and a significant increase was observed in the amniotic fluid index in second trimester.

**Conclusion:**

In Ramadan there was no bad fetal outcome between pregnant women with fasting and pregnant women without fasting. Pregnant women who want to be with fast, should be examined by doctors, adequately get breakfast before starting to fast and after the fasting take essential calori and hydration. More comprehensive randomized studies are needed to explain the effects of fasting on the pregnancy and fetal outcomes.

## INTRODUCTION

According to the Islamic religion, Muslims in the holy month of Ramadan does not eat and drink anything (fasting) from sunrise to sunset for a month. The duration of fasting vary from 9 hours to 18 hours according to season. According to Islam, pregnant women are exempt from fasting in the month of Ramadan. Most pregnant women do not fast during Ramadan because of not well-being and for the baby. On the other hand, despite several risk factors some pregnant women fast during Ramadan.^1^

During fasting several metabolic and physiological changes are observed in fasting people.^2^,^3^ In addition, there are limited number of studies that shows the effect of fasting in fetus and mother. While some studies reported that fasting have no effect to pregnancy, some reported alterations in fetal parameters.^2^,^4^,^5^ Previous studies were usually conducted in the pregnant women who were in third trimester of the gestation. Our study differs in that we evaluated the changes in all trimesters of pregnant women who fast during Ramadan and who do not fast.

## METHODS

We performed this study in Antakya State Hospital of Obstetrics and Child Care, between 28 June 2014 and 27 July 2014 (during the month of Ramadan). We followed all pregnant women for fetal outcomes until June 2014 from January 2015. A total of two hundred forty healthy pregnant women were included to this study groups. The three groups were divided according to the trimesters which equally consisted that pregnant women with / without fasting. The study protocol was approved by the institutional ethics committee. All subjects included in the study signed an informed consent. Ultrasonography was performed on all subjects in the beginning and then once a week until the end of Ramadan.

In the first trimester group, we evaluated the increase of fetal biparietal diameter (BPD) and reverse ‘a’ wave with ductus venosus Doppler. In the second and third trimester group, ultrasonography was performed to measure the following measurements: increase of BPD, increase of fetal femur length (FL), increase of estimated fetal body weight (EFBW), fetal biophysical profile (BPP), amniotic fluid index (AFI), and umbilical artery systole/diastole (UAD S/D) ratio. Fetal body weight was measured using Hadlock’s Formula.^6^ Amniotic fluid index was also calculated by the sum of deepest vertical pocket in four uterine quadrants measured in sonography. Oligohydramnios is defined as amniotic fluid index of 5 cm. To remove the effect of other factors causing oligohydramnios and polihydramnios, all cases with urinary or skeletal anomalies, intrauterine growth retardation, multiple pregnancy, diaphragmatic hernia, diabetes, fetal hydrops, and premature rupture of membrane, were excluded from the study. Flow velocimetry waveforms were obtained by Doppler ultrasonography as described previously.^7^ High definition image (HDI; A 3.5 MHz convex transducer, Applio-Toshiba, Otamara, Japan) was used to obtain AFI and Doppler waveforms. Subjects with the following conditions were excluded: diabetes, thyroid dysfunctions, Cushing syndrome, adrenal disease, preeclampsia, multiple pregnancies. Multivitamin, calcium (1 g/day) and iron (100 mg/day) supplementations were given to all subjects. All the subjects were advised to drink much water every night to prevent hypo-hydratation. In addition, the weight of newborns, apgar score and requirement of newborn intensive care (NICU) were recorded.

For statistical analysis, Means and standard deviations were used to describe numerical variables. The Kolmogorov-Smirnov test was used to evaluate the distribution pattern of the data. The Mann–Whitney U test was used to perform statistical comparisons between groups. Statistical significance was defined as p-value less than 0.05. Data analysis was obtained with SPSS for Windows 15.0 (Statistical Package for Social Sciences; SPSS Inc., Chicago, IL).

## RESULTS

A total of 240 patients (mean age 26.22±3.7) were enrolled to the study. The baseline characteristics of first trimester pregnant with and without fasting are shown in Table-I. There were no significant differences between the groups in terms of demographic characteristics of participants, fetal measurements and newborn features. The baseline characteristics of second trimester pregnants with and without fasting are shown in Table-II. There were no significant differences between the groups in terms of mean age, body mass index (BMI), gravidity, parity, gestastional age, increase of fetal BPD, increase of fetal EFBW, UAD S/D ratio, ratio of normal vaginal delivery/cesearen, newborn weight, low born weight, apgar score and requirement of newborn intensive care. However, increase of maternal weight and amniotic fluid index (AFI) mm values were significantly higher in pregnants without fasting as compared to pregnants with fasting. The baseline characteristics of second trimester pregnants with and without fasting are shown in Table-III. There were no significant differences between the groups in terms of demographic characteristics of participants, fetal measurements and newborn features. However, just increase of maternal weight values were significantly higher in pregnants without fasting as compared to pregnants with fasting.

**Table-I:** Demographic and clinical data of pregnant women in the first trimestere with/without fasting.

	Group 1 (With Fasting) (n=40)	Group 2 (Control) (n=40)	P value
Mean age	24.67±5.2	25.3±5.1	NS
BMI	21.2±2.3	20.8±3.1	NS
Gravidity	2.8 (1.8-2.9)	3.1(2.4-3.2)	NS
Parity	2.2(1.4-2.6)	2.4 (1.4-2.5)	NS
Gestastional age	14 ± 2.2	14±1.6	NS
Maternal weight increase	1.8(0.8–2.4)	2.1(1.3–3.1)	NS
Increase of Fetal BPD (mm)	3.4 (3.1–4.3)	3.6 (3.0-4.8)	NS
Reverse ‘a’ wave	3(%)	5(%)	NS
Ratio of NVD/CS	28/12	30/10	NS
Newborn weight	3015 ± 615.3	3052 ± 595.6	NS
LBW	2(5%)	1(2,5%)	NS
Apgar Score	1 Min7.82 ±0.50	1 Min7.70 ±0.40	NS
	5 Min9.40 ±0.35	5 Min9.50 ±0.30	NS
Requirement ofNICU	1(2,5%)	1(2,5%)	NS

BMI; Body Mass Index, BPD; Fetal Biparietal Diameter, NVD; Normal Vaginal Delivery, CS; Cesarean Section, LBW; Low Born Weight, NICU; Newborn Intensive Care Unit.

**Table-II:** Demographic and clinical data of pregnant women in the second trimestere with/without fasting.

	Group 1 (With Fasting) (n=40)	Group 2 (Control) (n=40)	P value
Mean age	26.46 ±4.3	27.26 ±3.9	NS
BMI	23.1±2.4	22.9±2.2	NS
Gravidity	3.4(1.8-3.2)	3.5(1.9-2.2)	NS
Parity	2.4(1.9-2.8)	3.2(2.2-2.6)	NS
Gestastional age	26.1 ± 2.3	26.4±1.4	NS
Maternal weight increase	3.4 (0.6–4.1)	4.4 (1.3–3.8)	0.03
Increase of Fetal BPD (mm)	3.7(3.1–4.8)	3.9(3.2-4.8)	NS
Increase of Fetal FL (mm)	3.4 (2.9–4.1)	3.6 (2.4–3.8)	NS
Increase of EFBW (g)	370(324–441)	390(355–486)	NS
AFI mm	11.4(9.8–14.1)	16.2(12.6–18.3)	0.02
Umblical artery S/D ratio	2.6(2.4–3.2)	2.4 (2.2–3.0)	NS
Ratio of NVD/CS	34/6	32/8	NS
Newborn weight	2925 ±542.4	3013±524.2	NS
LBW	1(2,5%)	1(2,5%)	NS
Apgar Score	1 Min7.64 ±0.60	1 Min7.72±0.35	NS
	Min8.92 ±0.40	5 Min9.1 ±0.25	NS
Requirement of NICU	-	-	-

BMI; Body Mass Index, BPD; Fetal Biparietal Diameter, FL fetal femur length, EFBW estimated fetal body weight, AFI amniotic fluid index, S/D systole/diastole ratio, NVD; Normal Vaginal Delivery, CS; Cesarean Section, LBW; Low Born Weight, NICU; Newborn Intensive Care Unit.

**Table-III:** Demographic and clinical data of pregnant women in the third trimestere with/without fasting.

	Group 1 (With Fasting) (n=40)	Group 2 (Control) (n=40)	P value
Mean age	27.53 ± 6.2	29.16 ±4.6	NS
BMI	22.5±3.3	21.5±2.4.1	NS
Gravidity	3.2 (2.1-3.4)	3.8(2.6-3.1)	NS
Parity	2.8 (1.8-3.2)	3.2 (2.1-3.4)	NS
Gestastional age	32 ± 2.2	31±1.8	NS
Maternal weight increase	3.0 (0.7–3.9)	4.2 (1.1–4.2)	0.02
Increase of Fetal BPD (mm)	3.9 (3.3–5.2)	4.1 (3.2-5.1)	NS
Increase of Fetal FL (mm)	3.6 (2.8–4.2)	3.7 (2.9–4.1)	NS
Increase of EFBW (g)	650(485–872)	670 (510–916)	NS
AFI mm	10.8(9.6–13.2)	12.4 (10.6–15.1)	NS
Umblical artery S/D ratio	2.1(1.8–3.1)	2.0 (1.9–2.8)	NS
Ratio of NVD/CS	29/11	31/9	NS
Newborn weight	3029 ±530.5	3045±572.6	NS
LBW	1(2,5%)	2(2,5%)	NS
Apgar Score	1 Min7.94 ±0.40	1 Min7.80±0.55	NS
	5 Min8.84 ±0.60	5 Min9.0 ±0.45	NS
Requirement of NICU	1	1	NS

BMI; Body Mass Index, BPD; Fetal Biparietal Diameter, FL fetal femur length, EFBW estimated fetal body weight, AFI amniotic fluid index, S/D systole/diastole ratio, NVD; Normal Vaginal Delivery, CS; Cesarean Section, LBW; Low Born Weight, NICU; Newborn Intensive Care Unit.

## DISCUSSION

Similar to previous studies, in our study, biometric and Doppler measurements have not showed a negative effect in terms of fetal outcomes between non-fasting and fasting pregnant women. On the other hand, a statistically significant increase was observed in maternal weight in the second and third trimesters and a significant increase was observed in the amniotic fluid index in second trimester.

The Ramadan is a holy month for the Muslims in which the duration of fasting changes depending on time zone and season and it lasts one month.^8^ In Ramadan, healthy adults (who reach puberty) do not eat or drink anything from sunrise to sunset.^9^,^10^

Fasting in Ramadan can lead to some changes in the body of fasting-people. These metabolic problems can be listed as; weight loss, reduction in systolic blood pressure and decrease in blood glucose.^11^,^12^ In addition to the physiological changes during pregnancy, fasting can cause more significant changes in pregnant women.

One of the important physiological changes in pregnancy is weight gain. In order to gain adequate weight enough calories should be taken. Caloric intake would be limited in pregnant women due to fasting. Therefore, weight gain may not be enough in fasting pregnant women. However, it was not shown that fasting have negative impact on women’s health.

One study has shown a significant reduction in the weight of fasting pregnant women in the third trimester.^13^ A similar study was shown that weight gain and calorie intake was reduced in fasting pregnant women, but this situation has been reported to have no negative effect to health of the pregnant women.^14^ In our study, while in the first trimester no significant changes was observed in terms of weight gain, in the second and third trimesters there was a significant difference between fasting and non-fasting pregnant women (4.4 - 3.4, 4.2 - 3.0, p=0.03, p=0.02, respectively).

Adequate uteroplacental blood flow during pregnancy is essential for fetal growth and well-being.^15^ The most important factors for the continuation of fetal well-being is to adequate nourishment of mother and adequate maternal placental blood flow.

The potential harm to fetus can be encountered during Ramadan fasting in which the fasting duration can be up to 16 hours in some seasons. Also, in the fasting mother, changes can be observed in the fetal development due low weight gain and calorie intake. A number of methods are used to determine whether fasting harm fetal development.^16^ Sonographic biometry measurements, uteroplacental and fetal vascular doppler parameters are used to determine the effect of maternal fasting on fetus. Previous studies have reported that maternal fasting have no significant effect to fetal biometric measurements^4^,^14^,^17^ and uterine artery and umbilical artery blood flow.^18^,^19^

In a study conducted on Somalian and Bangladeshi women, it was shown that maternal fasting have no impact on intrauterine growth and birth time.^20^ In the present study, fetal development and Doppler parameters were evaluated in fasting and non-fasting pregnant women in all trimesters. As a result, no significant adverse effect was observed in mothers. Also, in the second trimester, a significant reduction was observed in amniotic fluid index (16.2 - 11.4, p=0.02).

Neonatal anthropometric measurements in fasting pregnant women have not been clearly demonstrated. Newborn weight is the only realistic data that we have. Additionally, head circumference and height can also be used for the evaluation.

In a cross-sectional study conducted by Makvendi et al. no significant difference was reported between the fasting and non-fasting pregnant women in terms of neonatal anthropometric measurements.^21^ In another study, birth weight, head circumference, height and average thyroid hormone parameters were assessed in the third trimester. Similar to previous results, no significant difference was observed between fasting and non-fasting pregnant women.^22^

Our study results also showed that fasting do not lead to a significant reduction in the birth weight of infants. The most important limitations of our study are that the study population is small and single-centered.

## CONCLUSIONS

No adverse fetal effect was observed in the fasting pregnant women in contrast to non-fasting pregnant women during Ramadan. Pregnant women who want to fast should first contact her doctor. They should get enough calories and fluids before and after fasting. Large-scaled randomized studies are needed to clearly understand whether fasting have adverse effect to pregnancy and fetal development.
